# Molecular detection and phylogenetic characterization of *Babesia* and *Ehrlichia* in free-living opossums from southeastern Brazil

**DOI:** 10.1007/s11686-026-01325-x

**Published:** 2026-06-11

**Authors:** Stephany Rocha Ribeiro, Larissa Pereira Rodrigues, João Otávio Mochiuti, Catharina Eulasya Viana Resende, Gilberto Salles Gazeta, Ana Beatriz Borsoi, Mariana Guimarães Côrtes da Cruz, Karla Bitencourth, Rodrigo Hidalgo Friciello Teixeira, Lígia Souza Silveira da Lima

**Affiliations:** 1 Department of Genetics, Microbiology and Immunology, Institute of Biosciences, São Paulo State University (UNESP), Botucatu, São Paulo, Brazil; 2 School of Veterinary Medicine and Animal Science, São Paulo State University “Júlio de Mesquita Filho”, Botucatu, São Paulo, Brazil; 3 Laboratory of Ticks and Other Apterous Arthropods, Collection of Apterous Arthropod Vectors of Community Health Importance, Oswaldo Cruz Institute (Fiocruz), Rio de Janeiro, Brazil; 4 Municipal Zoological Park “Quinzinho de Barros”, Sorocaba, São Paulo, Brazil; 5 Sorocaba University, Sorocaba, São Paulo, Brazil; 6 Postgraduate Program in Wild Animals, São Paulo State University “Júlio de Mesquita Filho” (UNESP), Botucatu, São Paulo, Brazil

**Keywords:** *Didelphis*, *Hepatozoon*, Marsupials, One health, Vector-borne infections, Wildlife

## Abstract

**Purpose:**

This study investigated the occurrence of vector-borne pathogens in free-living opossums (*Didelphis albiventris* and *Didelphis aurita*) from São Paulo State, Brazil, with emphasis on *Babesia* spp., *Hepatozoon* spp., and *Ehrlichia* spp.

**Methods:**

Blood samples from 115 opossums (95 *D. albiventris* and 20 *D. aurita*) were analyzed using parasitological and molecular approaches. Conventional PCR assays were performed for *Babesia* spp. and *Hepatozoon* spp., whereas nested PCR was used for *Ehrlichia* spp. Positive amplicons were sequenced and subjected to phylogenetic analysis.

**Results:**

Five samples (4.35%) from *D. albiventris* collected in Botucatu were positive for *Babesia* spp. based on the 18 S rRNA gene, and two samples (1.74%) were positive for *Ehrlichia* spp. based on the *dsb* gene. No *Hepatozoon* spp. DNA was detected. Sequence analysis revealed 100% identity between the *Babesia* amplicons and *Babesia* sp. (GenBank: MW290046), while one *Ehrlichia* sequence showed 100% identity with *Ehrlichia* sp. strain Natal (GenBank: KY207546; OP270482), and the second sequence showed 99.71% identity with *Ehrlichia* sp. (GenBank: OP270482). Phylogenetic reconstruction clustered the *Babesia* sequences within the South American Marsupialia Group and positioned the *Ehrlichia* sequences together with lineages previously reported in South American marsupials.

**Conclusion:**

These findings provide the first molecular detection of *Babesia* sp. and *Ehrlichia* sp. in free-living *D. albiventris* from Botucatu, São Paulo State, Brazil. The results expand current knowledge on vector-borne pathogens in synanthropic marsupials and reinforce the importance of continued surveillance to improve understanding of host–pathogen interactions at urban–wildlife interfaces within a One Health perspective.

## Introduction

Environmental changes driven by anthropogenic activities, such as unplanned urban expansion and habitat destruction, can alter the geographic distribution of disease vectors and consequently affect the epidemiology of infectious diseases [1–3]. As a result, wild animals increasingly occupy urban and peri-urban environments in search of food and shelter, intensifying contact with domestic animals and humans and increasing the risk of zoonotic pathogen transmission [3].

Opossums are synanthropic marsupials of the genus *Didelphis* (family Didelphidae, order Didelphimorphia). Of the six species described in the Americas, four occur in Brazil: *Didelphis albiventris*, *Didelphis aurita*, *Didelphis imperfecta*, and *Didelphis marsupialis* [4]. Due to environmental changes and their ecological plasticity, these animals increasingly occupy peri-urban and urban environments, where they exploit anthropogenic resources such as food, shelter, and water, frequently resulting in their rescue and admission to rehabilitation centers [4, 5]. These animals play important ecological roles as seed dispersers, scavengers, and controllers of pest populations, contributing to ecosystem balance [4, 5]. In addition, they participate in the epidemiology of several parasitic diseases, acting as reservoirs of a wide range of pathogens, including arthropods, helminths, and protozoa [4, 6, 7].

Among these pathogens are the protozoa *Babesia* spp. and *Hepatozoon* spp., as well as bacteria of the genus *Ehrlichia*, which are etiological agents of babesiosis, hepatozoonosis, and ehrlichiosis, respectively [7–9]. These agents are vector-borne pathogens maintained through distinct ecological routes involving different arthropod species and host interactions. Their occurrence in synanthropic wildlife may contribute to complex transmission networks at human–animal–environment interfaces [8]. *Babesia* spp. are intraerythrocytic protozoa of the order Piroplasmida, which parasitize the erythrocytes of vertebrate hosts, and are transmitted through the inoculation of sporozoites during tick feeding [9–14]. In contrast, *Hepatozoon* spp. infect a wide range of vertebrate hosts and are typically transmitted through the ingestion of infected arthropods containing oocysts, although transmission may also occur via predation on infected intermediate hosts [15–18]. *Ehrlichia* spp. are obligate intracellular Gram-negative bacteria of the family Anaplasmataceae, with recognized zoonotic potential, that infect mammalian leukocytes and are transmitted during the blood meal of infected ticks [9, 19, 20]. In Brazil, the occurrence of *Babesia*, *Hepatozoon*, and *Ehrlichia* in opossums has been reported in several studies, highlighting their role in the maintenance of vector-borne pathogens across different ecosystems [21–30].

Therefore, the present study aimed to investigate the occurrence of *Babesia* spp., *Hepatozoon* spp., and *Ehrlichia* spp. in blood samples from free-living white-eared opossums (*Didelphis albiventris*) and black-eared opossums (*Didelphis aurita*) from São Paulo State, Brazil, using parasitological and molecular approaches.

## Materials and methods

### Ethics approval

All procedures performed in this study were conducted in accordance with applicable ethical standards and were approved by the Animal Use Ethics Committee (CEUA) of São Paulo State University (UNESP) under protocols 0237/2022 and 0055/2021. In addition, authorization for wildlife sampling was granted by the Biodiversity Authorization and Information System (SISBIO) under permit numbers 84319-1 and 78287-1.

### Study area

**Fig. 1:**
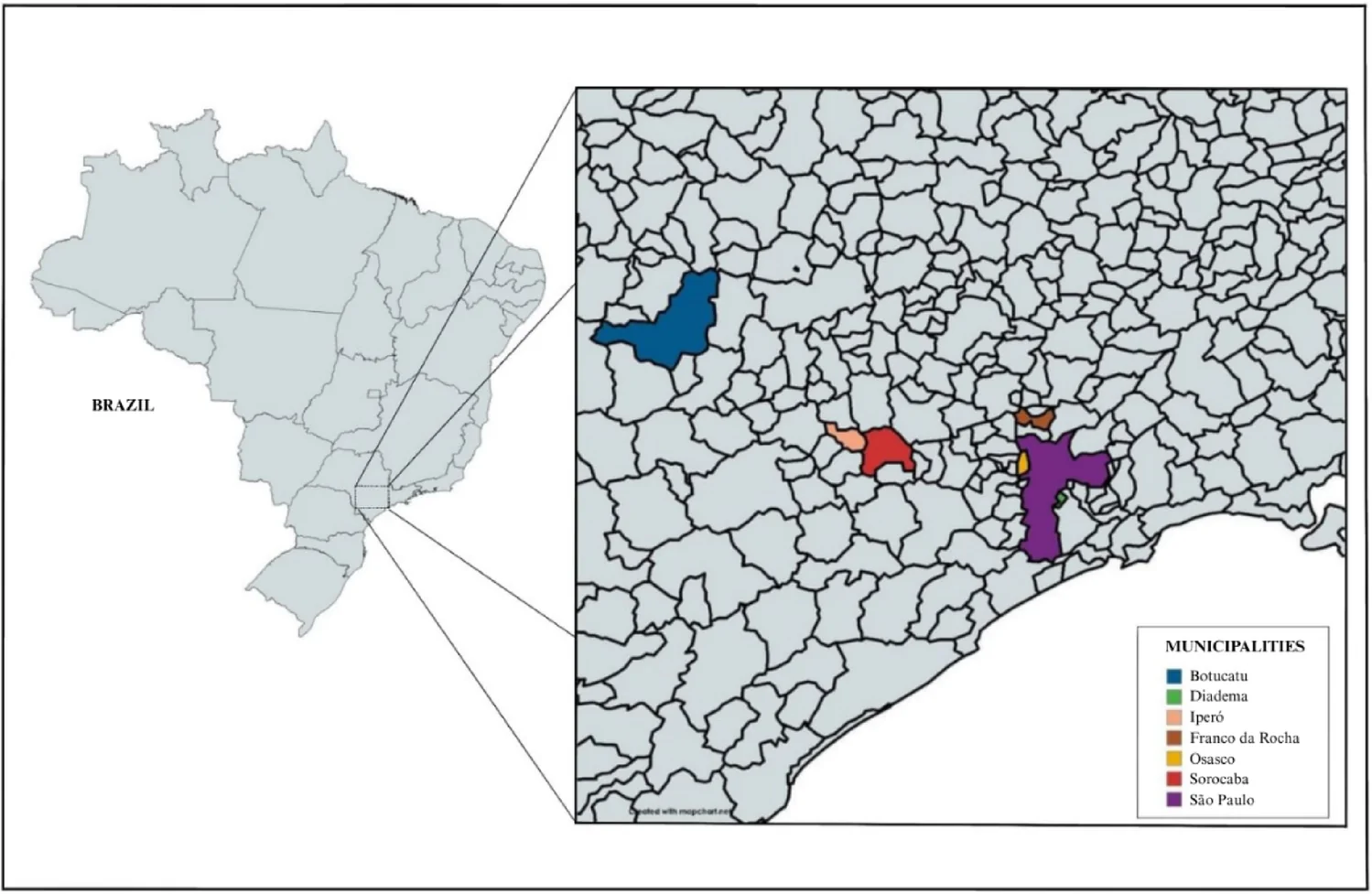
Geographical locations of the seven municipalities in São Paulo State, Brazil, where opossums (*Didelphis albiventris* and *Didelphis aurita*) were sampled

The study was conducted in three institutions located in São Paulo State, Brazil: the Municipal Zoological Park “Quinzinho de Barros” (PZMQB), in Sorocaba; the Wildlife Management and Conservation Center (CeMaCAS), in São Paulo; and the Environmental Health Surveillance Division (VAS) in Botucatu. The sampled animals originated from seven municipalities within São Paulo State: Botucatu, Diadema, Franco da Rocha, Iperó, Osasco, São Paulo, and Sorocaba (Fig. 1).

### Sample and ectoparasite collection

Samples were collected from June 2021 to May 2023. Upon admission to the facilities, animals were physically restrained using appropriate safety equipment by trained personnel. Blood samples were collected from the jugular, femoral, or lateral coccygeal veins following clinical evaluation by a licensed veterinarian. Approximately 1.5 mL of whole blood was obtained using 24G or 22G needles and 3 mL syringes. Samples were transferred to microtubes containing EDTA and stored at − 20 °C until DNA extraction.

During clinical examination a subset of opossums was found to be infested with ectoparasites, possibly due to grooming behavior and feeding habits, as these animals are known to consume fleas and ticks [4]. Ectoparasites were manually removed using fine-tipped tweezers and preserved in 70% ethanol for subsequent morphological identification. Whenever possible, specimens were identified based on morphological characteristics [31, 32]. Molecular screening of ectoparasites was not performed because collection was opportunistic and the number of recovered specimens was insufficient to support additional analyses.

### Microscopic analysis

Blood smears were prepared for morphological evaluation of hemoparasites. Whole blood samples collected during clinical procedures were centrifuged, and smears were prepared from the buffy coat fraction using the wedge technique. After air-drying, slides were stained using a commercial rapid Panoptic kit (Newprov Laboratory Products Ltda, PA205, Brazil), according to the manufacturer’s instructions.

Each slide was examined under a light microscope using a 100× oil immersion objective for the investigation of blood-associated microorganisms and cellular inclusions. Blood smears from PZMQB animals were prepared at the Cão Boy Veterinary Clinic Laboratory (Sorocaba, SP), whereas those from CeMaCAS animals were prepared at the Laboratório de Estudos da Fauna Silvestre (São Paulo, SP). This procedure was not performed for samples from VAS, as only frozen genomic DNA was available.

### Molecular analysis

DNA extraction and quantification were performed at the Animal Genetics Laboratory, Department of Genetics, Microbiology and Immunology, Institute of Biosciences, São Paulo State University (UNESP), Botucatu, SP, Brazil. PCR amplification, amplicon purification, and sequencing were performed at the Laboratory of Ticks and Other Apterous Arthropods, Oswaldo Cruz Institute, Rio de Janeiro, RJ, Brazil.

Genomic DNA was extracted using the phenol–chloroform method described by Sambrook and Russell [33]. Any procedural adaptations were restricted to routine laboratory practices and did not affect the methodological principles of the original protocol. DNA concentration and purity were determined using a NanoDrop ND-1000 spectrophotometer (Thermo Fisher Scientific), and DNA integrity was assessed by electrophoresis on 1.5% agarose gel stained with GelRed (0.1 µL/10 mL; Uniscience) in 1× TAE buffer.

Conventional PCR assays were performed to detect *Babesia* spp. and *Hepatozoon* spp., whereas nested PCR was used for *Ehrlichia* spp., using a Loccus^®^ AmpliGene Lite thermal cycler. Primer sequences, expected amplicon sizes, and corresponding references are listed in Table 1.

**Table 1:** Primer sets used for amplification of target DNA fragments of *Babesia* spp., *Hepatozoon* spp. and *Ehrlichia* spp

Agent	Target Gene	Primer pair	Primer sequence (5′–3′)	Amplified size(bp)	Reference
*Babesia* spp.	18 S rRNA	BAB143-167 BAB858-834	F– CCGTGCTAATTGTAGGGCTAATACA R– CCTCTGACAGTTAAATACGAATGCC	715 bp	Lemos et al. [34]
*Hepatozoon* spp.	18 S rRNA	HEP-F HEP-R	F– GGTAATTCTAGAGCTAATACATGAGC R– ACAATAAAGTAAAAAACAYTTCAAAG	574 bp	Almeida [35]
*Ehrlichia* spp.	dsb	DSB-330 DSB-380 DSB-720	F– GATGATGTTTGAAGATATSAAACAAAT F– ATTTTTAGRGATTTTCCAATACTTGG R– CTATTTTACTTCTTAAAGTTGATAWATC	401 bp 349 bp	Doyle et al. [36] Almeida [35]

*bp* Base pairs,* F* Forward primer,* R* Reverse primer

 Amplification of *Babesia* spp., targeted a 715 bp fragment of the 18 S rRNA gene using primers BAB143–167 F and BAB858–834R, and cycling conditions followed those described by Lemos et al. [34]. Amplification of *Hepatozoon* spp. targeted a 574 bp fragment of the 18 S rRNA gene using primers Hep144–169 F and Hep743–718R under conditions previously described by Almeida [35]. Detection of *Ehrlichia* spp. was performed by nested PCR targeting the *dsb* gene. using primers DSB-330 F/DSB-720R (401 bp) in the first reaction and DSB-380 F/DSB-720R (349 bp) in the second reaction, following amplification conditions previously described by Almeida [35] and Doyle et al. [36]. PCR mixtures were prepared according to the original protocols [34–36]. Positive controls included DNA from *Babesia vogeli*, *Hepatozoon canis*, and *Ehrlichia* spp., while nuclease-free water was used as a negative control.

Amplified products were visualized by electrophoresis on 2% agarose gels in 1× TAE buffer at 90 V for 50 min. Gels were stained with GelRed and visualized under UV light using a UV transilluminator (Cambridge, UK) with UVP^®^ VisionWorksLS™ software. Samples presenting bands of the expected size were considered positive.

PCR products were purified using the ReliaPrep™ DNA Clean-Up and Concentration System (Promega) and subjected to bidirectional sequencing using an ABI 3730xl DNA Analyzer (Applied Biosystems) and the BigDye Terminator v3.1 Cycle Sequencing Kit.

Sequences were edited in Chromas Pro v1.5 (Technelysium Pty Ltd., Australia) and compared with GenBank entries using BLASTn [37]. Multiple sequence alignment was performed using ClustalW. Phylogenetic analyses were conducted using the maximum likelihood (ML) method implemented in PHYML v3.0 [38]. The best-fit evolutionary models were selected based on the Bayesian Information Criterion (BIC) in MEGA v6.0 (*Babesia*: GTR + G+I; *Ehrlichia*: T92 + I). Node support was evaluated using the approximate likelihood ratio test (aLRT) with 1,000 replicates. *Cardiosporidium cionae* and *Ehrlichia muris* were included as outgroups for the *Babesia* and *Ehrlichia* phylogenetic reconstructions, respectively.

## Results

### Sample collection

A total of 115 blood samples were obtained from free-living opossums admitted to the Municipal Zoological Park “Quinzinho de Barros” (PZMQB), Wildlife Management and Conservation Center (CeMaCAS), and Environmental Health Surveillance Division (VAS), regardless of health status (Table 2). The sample comprised 95 *D. albiventris* and 20 *D. aurita*. Of the total animals sampled, 33 (28.70%) were males, 58 (50.43%) were females, and 24 (20.87%) were of indeterminate sex. Regarding age class, 16 (13.91%) were classified as joeys, 16 (13.91%) were juveniles, and 83 (72.18%) were adults.

**Table 2:** Distribution of free-living opossums (*Didelphis albiventris* and *Didelphis aurita*) sampled according to institution of origin in São Paulo State, Brazil

Institution	Species	*n* per species	Total per institution
PZMQB	*Didelphis albiventris*	29	32
	*Didelphis aurita*	3	
CeMaCAS	*Didelphis albiventris*	4	21
	*Didelphis aurita*	17	
VAS	*Didelphis albiventris*	62	62
	*Didelphis aurita*	0	
Total		115	

### Ectoparasites

Among the animals from the PZMQB (Sorocaba), six opossums were infested with fleas identified as *Ctenocephalides felis*, two with ticks identified as *Amblyomma longirostre*, and one harbored second-instar larvae of *Cochliomyia hominivorax*. At CeMaCAS (São Paulo), two opossums were parasitized by fleas, one by ticks, and one by a mite. At VAS (Botucatu), six opossums were infested with fleas and two with ticks. Morphological identification of ectoparasites from CeMaCAS and VAS was not performed, and no molecular analyses were conducted on these specimens.

### Microscopic analysis

Microscopic examination of blood smears revealed intraleukocytic inclusions morphologically suggestive of *Ehrlichia*-like organisms in one individual (sample 28), an adult female *D. aurita* from PZMQB, Sorocaba. No protozoan parasites were detected by microscopy. The sample presenting these inclusions tested negative by PCR, whereas the PCR-positive samples for *Ehrlichia* spp. did not show visible inclusions in blood smears. Therefore, microscopic findings alone were not considered sufficient to confirm infection.

### Molecular analysis

DNA extraction yielded measurable concentrations in all 115 samples (minimum concentration: 6.1 ng/µL). DNA integrity was assessed by agarose gel electrophoresis prior to molecular analyses.

Five samples (4.35%), from *D. albiventris* collected in Botucatu, São Paulo, were positive for *Babesia* spp. by PCR amplification of the 18 S rRNA gene (Table 3).

**Table 3:** BLASTn results of the nucleotide sequences obtained for *Babesia* sp. and *Ehrlichia* sp. detected in white-eared opossums (*Didelphis albiventris*) from Botucatu, São Paulo, Brazil

Agent	Sample ID	Molecular marker	Identity (%)	Closest GenBank sequence (accession no.)	Query cover (%)	E-value
*Babesia* sp.	BAB 94	18 S rRNA	100	*Babesia* sp. (MW290046)	100	0.0
*Babesia* sp.	BAB 102	18 S rRNA	100	*Babesia* sp. (MW290046)	100	0.0
*Babesia* sp.	BAB 103	18 S rRNA	100	*Babesia* sp. (MW290046)	100	0.0
*Babesia* sp.	BAB 109	18 S rRNA	100	*Babesia* sp. (MW290046)	100	0.0
*Babesia* sp.	BAB 114	18 S rRNA	100	*Babesia* sp. (MW290046)	100	0.0
*Ehrlichia* sp.	ER 66	*dsb*	100	*Ehrlichia* sp. (KY207546; OP270482)	100	3e−167
*Ehrlichia* sp.	ER 111	*dsb*	99.71	*Ehrlichia* sp. (OP270482)	100	2e−179

**Fig. 2:**
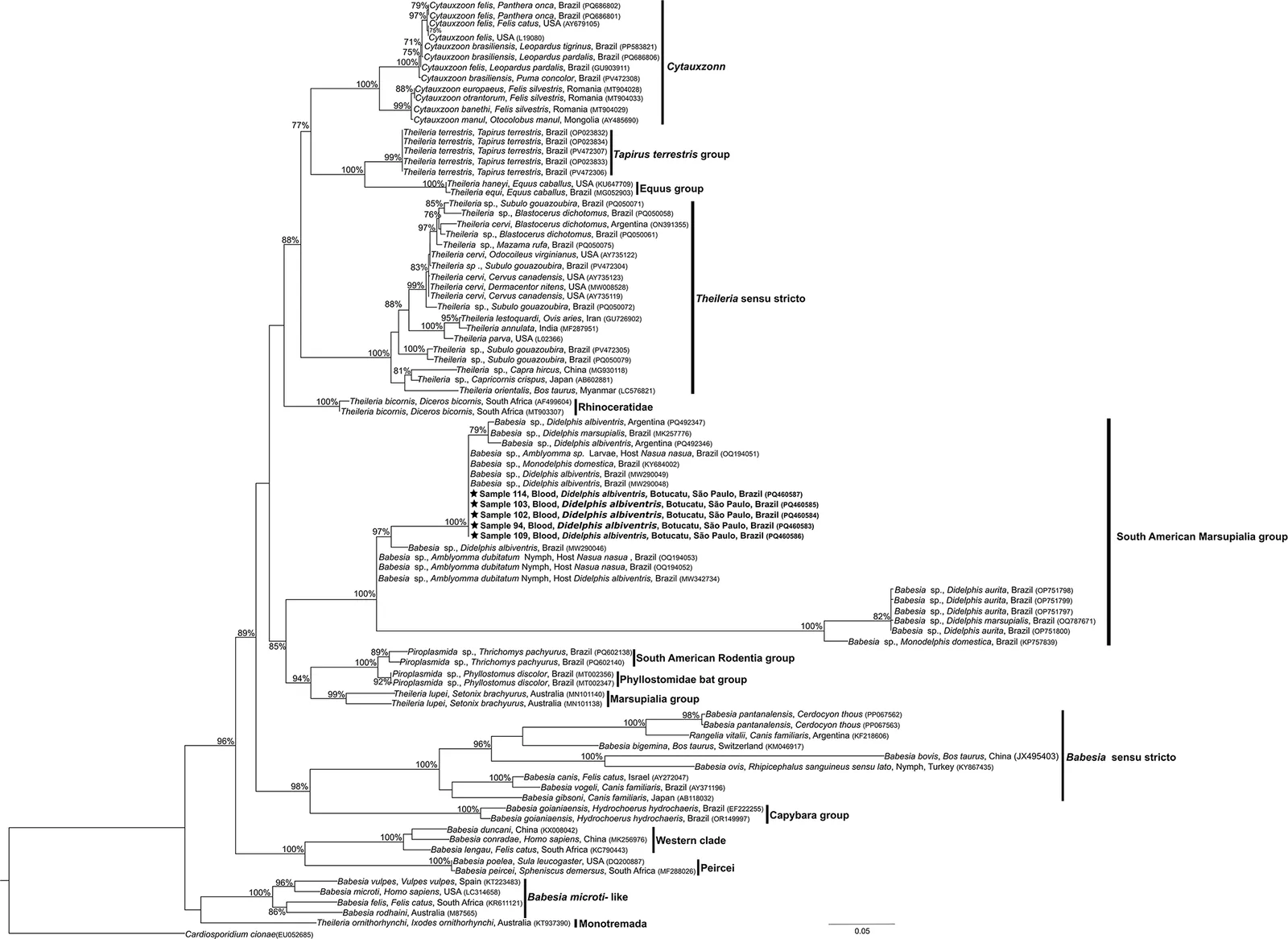
Phylogenetic tree inferred by the maximum likelihood method using the GTR + G+I evolutionary model, based on partial sequences of the 18 S rRNA gene (1771 bp) of piroplasmids. Sequences of *Babesia* sp. obtained from *Didelphis albiventris* in Botucatu, São Paulo, Brazil, are indicated with a star. Numbers on branches represent approximate likelihood ratio test (aLRT) support values (cut-off 70%). *Cardiosporidium cionae* was used as the outgroup

BLASTn analysis revealed that all five sequences shared 100% identity with *Babesia* sp. (GenBank accession number MW290046), previously reported in *D. albiventris* from Brazil. Phylogenetic reconstruction based on the 18 S rRNA gene (1,771 bp) clustered these sequences (BAB 94, BAB 102, BAB 103, BAB 109 and BAB 114) within the South American Marsupialia clade, with strong statistical support (aLRT = 100%) (Fig. 2). Within this clade, the detected sequences formed a well-supported branch together with *Babesia* sp. previously identified in *D. albiventris*, *Monodelphis domestica* and *Amblyomma* sp. larvae collected from *Nasua nasua* in Brazil.

**Fig. 3:**
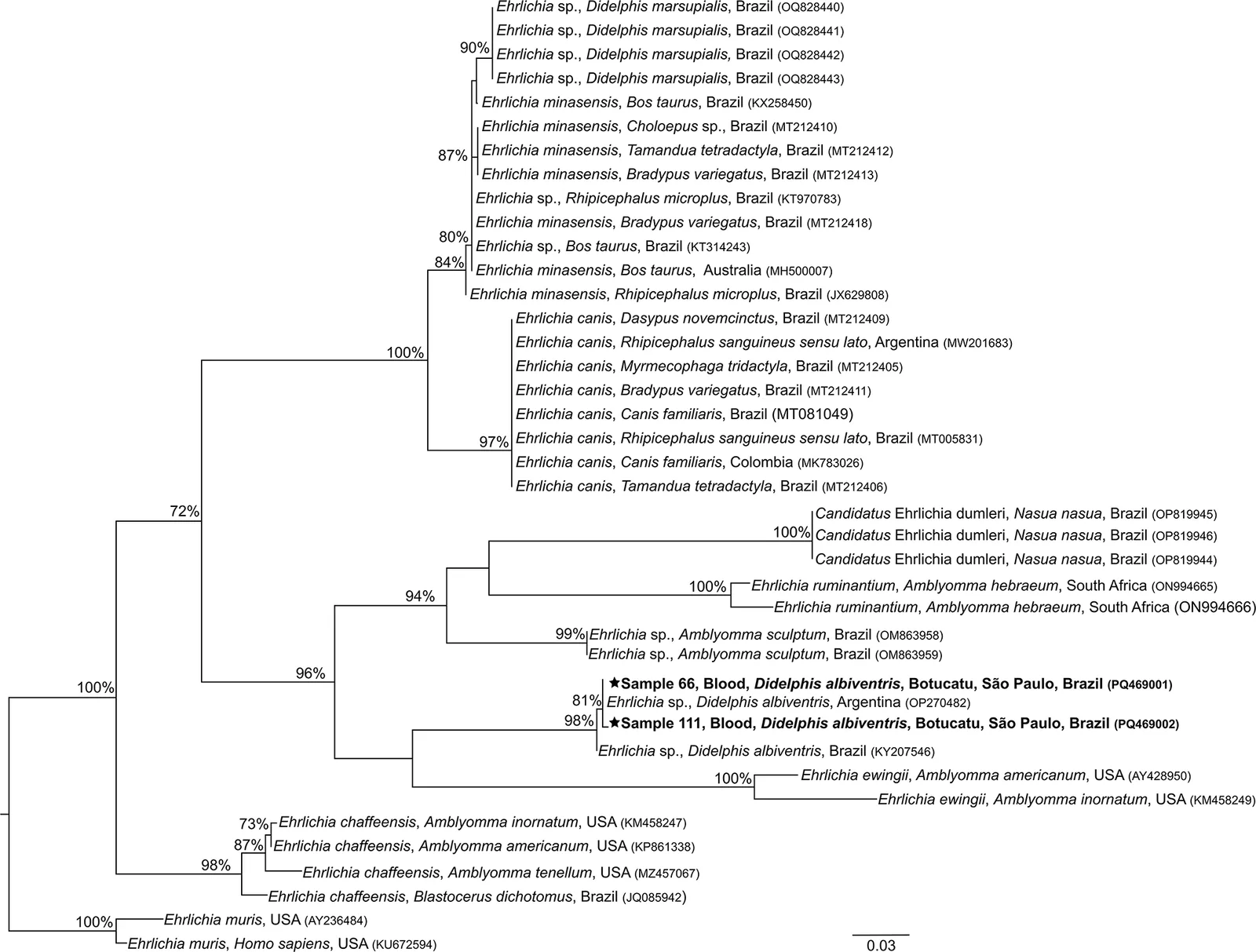
Phylogenetic tree inferred by the maximum likelihood method using the T92 + I evolutionary model, based on partial sequences of the disulfide oxidoreductase (*dsb*) gene (325 bp) of *Ehrlichia* spp. Sequences obtained from *Didelphis albiventris* in Botucatu, São Paulo, Brazil, are indicated with a star. Numbers on branches represent approximate likelihood ratio test (aLRT) support values (cut-off 70%) *Ehrlichia muris* was used as the outgroup

Additionally, two samples (1.74%) from *D. albiventris* collected in Botucatu, São Paulo, were positive for *Ehrlichia* spp. by nested PCR targeting the *dsb* gene (Table 3). BLASTn analysis showed that one sequence (ER 66) shared 100% identity with *Ehrlichia* sp. (GenBank accession numbers KY207546 and OP270482), whereas the second sequence (ER 111) showed 99.71% identity with *Ehrlichia* sp. (GenBank accession number OP270482). Phylogenetic analysis based on the *dsb* gene (325 bp) clustered these sequences within a clade comprising *Ehrlichia* sp. previously reported in *D. albiventris* from Brazil and Argentina, with high statistical support (aLRT = 98%) (Fig. 3).

No amplification of *Hepatozoon* spp. DNA was observed in any of the analyzed samples, despite successful amplification of the remaining molecular targets.

All molecularly positive samples originated from opossums collected in Botucatu, São Paulo.

## Discussion

Although intraleukocytic inclusions morphologically suggestive of *Ehrlichia*-like organisms were observed in one blood smear (sample 28, *D. aurita* from PZMQB, Sorocaba), molecular assays did not confirm infection. Therefore, these microscopic findings were interpreted cautiously and were not considered sufficient to establish infection status.

The absence of detectable hemoparasites in blood smears is consistent with previous studies reporting the limited sensitivity of parasitological methods, particularly in infections characterized by low or intermittent parasitemia, such as babesiosis and hepatozoonosis [9–14, 17, 39].

Interpretation of the microscopic findings should also consider methodological limitations, since parasitological evaluation was not available for all molecularly analyzed samples. In particular, samples from VAS consisted exclusively of frozen genomic DNA, precluding direct comparison between microscopic and molecular findings. These differences in sample availability may have influenced diagnostic sensitivity and reduced agreement between detection approaches.

The detection of *Babesia* and *Ehrlichia* in free-living opossums reinforces the role of synanthropic marsupials as components of complex host–pathogen systems in anthropized environments. Although the prevalence observed in the present study was relatively low, the detection of both agents exclusively in *D. albiventris* from Botucatu suggests spatial heterogeneity in pathogen occurrence and supports the importance of local ecological conditions in shaping transmission dynamics.

Previous reports from São Paulo State and other Brazilian regions provide additional context for interpreting these findings. Serological evidence of *Ehrlichia* spp. has previously been reported in *D. albiventris* and *D. aurita* from municipalities overlapping those investigated in the present study [21], while molecular detection of *Hepatozoon* spp. has also been described in opossums from Botucatu [22]. In contrast, no *Hepatozoon* DNA was detected in our samples, which may reflect differences in sampling strategy, temporal variation, or methodological sensitivity.

Similar patterns have been reported in other Brazilian biomes, where marsupials have been shown to harbor distinct hemoparasite assemblages [23, 26, 27, 30]. Together, these findings reinforce that pathogen occurrence in opossums appears to be influenced by ecological context, host exposure, and local vector communities rather than by a uniform distribution pattern.

Phylogenetic analysis confirmed that the *Babesia* sequences detected in *D. albiventris* from Botucatu clustered within the South American Marsupialia Group, as previously described [40] and further supported by studies conducted in Brazil and Argentina [28, 41, 42]. The clustering of all sequences within this group supports previous evidence of the association between *Didelphis* spp. and this lineage across South America.

Similarly, the *Ehrlichia* sequences obtained were positioned within the clade corresponding to *Ehrlichia* sp. strain Natal, previously described in northeastern Brazil and later detected in Argentina [25, 43]. This finding may indicate a broader geographic distribution of this genotype than currently documented and supports continued investigation of its occurrence in anthropized environments.

Although the 16 S rRNA gene is commonly employed for broader phylogenetic inference within Anaplasmataceae, the *dsb* gene was selected because it has been widely used for the molecular detection and comparative analysis of *Ehrlichia* spp., including studies involving wildlife hosts [25, 29, 36, 43]. Since the present study aimed to investigate pathogen occurrence and phylogenetic positioning rather than formal taxonomic characterization, the selected marker was considered appropriate. Nevertheless, the use of a single genetic marker may limit taxonomic resolution, and future studies incorporating additional loci, such as 16 S rRNA, *groEL*, and *omp-1*, may contribute to a more comprehensive characterization of these genotypes.

The detection of both *Babesia* and *Ehrlichia* in opossums from the same geographic area may reflect the circulation of multiple vector-borne agents within local wildlife communities. Although the transmission routes and vector species involved were not investigated in the present study, these findings highlight the need for future studies addressing vector competence, host–parasite interactions, and pathogen circulation dynamics, particularly in urban and peri-urban environments.

The exclusive detection of positive animals in Botucatu may suggest localized circulation of these pathogens, potentially influenced by environmental and ecological factors such as habitat alteration, host availability, and vector occurrence. However, the limited number of positive samples and the absence of *Hepatozoon* spp. detection warrant caution in interpreting geographic patterns and reinforce the need for broader temporal and spatial investigations to better understand infection dynamics in opossum populations.

Overall, the findings expand current knowledge on the occurrence and distribution of *Babesia* and *Ehrlichia* lineages in Brazil and support the potential involvement of *D. albiventris* in the maintenance of vector-borne pathogens. These results emphasize the importance of monitoring synanthropic marsupials and their ectoparasites at urban–wildlife interfaces under a One Health framework. Given the ecological characteristics of these hosts and their frequent occurrence in anthropized environments, continued surveillance may contribute to a better understanding of pathogen circulation and wildlife–human interfaces. Further studies are needed to investigate transmission dynamics, vector associations, and the epidemiological relevance of these agents in urban and peri-urban settings.

## Conclusion

This study expands current knowledge of vector-borne pathogens in opossums from southeastern Brazil and provides the first molecular evidence of *Babesia* sp. and *Ehrlichia* sp. in free-living *D. albiventris* from the municipality of Botucatu, São Paulo State. The *Babesia* sequences identified clustered within the South American Marsupialia Group, supporting previous evidence of a piroplasmid lineage associated with Neotropical marsupials. The detection of *Ehrlichia* sp. in the same host species, together with previous reports from Brazil and neighboring countries, suggests the circulation of this genotype in anthropized environments. These fundings reinforce the importance of continued surveillance of synanthropic wildlife and contribute to a better understanding of host–pathogen interactions at urban–wildlife interfaces within a One Health perspective.

## Data Availability

The data generated and analyzed during this study are available from the corresponding author upon reasonable request.
